# Optimization of Biosorptive Removal of Dye from Aqueous System by Cone Shell of Calabrian Pine

**DOI:** 10.1155/2014/138986

**Published:** 2014-10-21

**Authors:** Fatih Deniz

**Affiliations:** Birecik Anatolian High School, 63400 Birecik, Turkey

## Abstract

The biosorption performance of raw cone shell of Calabrian pine for C.I. Basic Red 46 as a model azo dye from aqueous system was optimized using Taguchi experimental design methodology. L_9_ (3^3^) orthogonal array was used to optimize the dye biosorption by the pine cone shell. The selected factors and their levels were biosorbent particle size, dye concentration, and contact time. The predicted dye biosorption capacity for the pine cone shell from Taguchi design was obtained as 71.770 mg g^−1^ under optimized biosorption conditions. This experimental design provided reasonable predictive performance of dye biosorption by the biosorbent (*R* ^2^: 0.9961). Langmuir model fitted better to the biosorption equilibrium data than Freundlich model. This displayed the monolayer coverage of dye molecules on the biosorbent surface. Dubinin-Radushkevich model and the standard Gibbs free energy change proposed physical biosorption for predominant mechanism. The logistic function presented the best fit to the data of biosorption kinetics. The kinetic parameters reflecting biosorption performance were also evaluated. The optimization study revealed that the pine cone shell can be an effective and economically feasible biosorbent for the removal of dye.

## 1. Introduction

Synthetic dyes are extensively used in many industrial applications including textile, leather, food processing, dyeing, cosmetics, paper, and dye manufacturing industries [1]. The release of various harmful dyes from these industries into the environment has attracted great attention worldwide in recent years. Dyes usually have a synthetic origin and complex chemical structure that make them persistence to light, oxidation, and biodegradable process. As is well known, the presence of dyes in water sources can cause reduction of light penetration, photosynthetic activity, and gas solubility in addition to visual pollution. Also many dyes and their degradation derivatives are highly toxic and carcinogenic [2]. It is necessary to remove these harmful dyes from contaminated water for a better ecosystem quality.

Biosorption is a very effective dye removal technique and now it is noted to be superior to other methods for water treatment with regard to ease of operation, cost economics, ecocompatibility, high efficiency, simplicity of design, and insensitivity to toxic substances [3, 4]. A considerable number of low-cost biosorbents have been recently applied for removal of dyes [5–7]. As compared to activated carbon, most of these materials have low biosorption potential. Thus, the search for excellent and efficient biosorbent is still going on [8]. Calabrian pine (*Pinus brutia* Ten.) is a characteristic species of the eastern Mediterranean. Its forests represent about 27% of the country's forest area, which totals at 5,854,673 ha in 2012 [9]. Pine tree cones are produced in large quantities at forest industries as a litter. These forest residues are potential lingo-cellulosic biomaterials for dye biosorption. They are very cheap, renewable, and of great availability. New usage as biosorbent of these materials is an attractive alternative from both environmental and economic aspects.

Design of experiment methodologies can be employed to minimize the number of experiments, time, and research costs. Artificial neural network (ANN) and genetic algorithm (GA) are well-known methods for multifactor process optimization. On the other hand, Taguchi experimental design is a simple and efficient tool for optimization of process [10]. Taguchi design can be used for process optimization more economically [11]. In this method, experiments are designed according to the orthogonal array technique. An orthogonal array is a fractional factorial design with pairwise balancing property. Using orthogonal array design can estimate how multiple process factors affect the performance characteristic simultaneously while minimizing the number of experiments [12].

The main objective of this study is to optimize the biosorption performance of raw cone shell of Calabrian pine for C.I. Basic Red 46 as a model azo dye from aqueous system using Taguchi experimental design methodology. The isotherm models of Freundlich, Langmuir, and Dubinin-Radushkevich were used for the equilibrium data analysis. The kinetic data were analyzed using the pseudo-first-order, pseudo-second-order, logistic, and intraparticle diffusion models. Besides, the relationship between the kinetic parameters and the biosorption performance was investigated. Finally, a single-stage batch biosorption system design for the dye removal was outlined based on the equilibrium data obtained.

## 2. Materials and Methods

### 2.1. Biosorbent Characterization

An infrared analysis was performed within the range 650–4000 cm^−1^using a Fourier Transform Infrared (FTIR) Spectrometer (Spectrum 100, PerkinElmer, USA) to identify the functional groups present on the pine cone shell. Besides, a Scanning Electron Microscope (SEM) (JSM-6390, JEOL, USA) was utilized to disclose the surface morphology of the biosorbent.

### 2.2. Preparation of Biosorbent and Dye Solution

The pine cone shells were collected from a plantation in Gaziantep, Turkey. After washing with distilled water to eliminate dust and other residues, the shells were dried at 80°C and then crushed, milled, and sieved. The fractions of particle between 63 and 500 *μ*m were selected for biosorption studies. These were then stored in an airtight plastic container to use as biosorbent without any further pretreatments.

As a model azo dye, C.I. Basic Red 46 was obtained from a local source. It was of commercial quality and used without further purification. A stock dye solution at a concentration of 500 mg L^−1^ was prepared by dissolving appropriate amount of the dye in distilled water. The experimental concentrations were obtained by the dilution of this solution. The pH values of working solutions were adjusted by the addition of 0.1 M HCl and 0.1 M NaOH solutions whenever necessary.

### 2.3. Experimental Setup

Taguchi experimental design (L_9 _(3^3^) orthogonal array) was used to optimize the dye biosorption by the pine cone shell. The selected factors and their levels for this biosorption study were biosorbent particle size (63–125, 125–250, and 250–500 *μ*m), dye concentration (40, 60, and 100 mg L^−1^), and contact time (30, 75, and 120 min). In order to investigate the effect of these experimental factors on the dye biosorption, batch biosorption experiments were carried out with 0.05 mg of the biosorbent with 50 mL of dye solutions of desired concentration at pH 8 in a series of 100 mL conical flasks. The samples were agitated at a constant speed in a temperature-controlled water bath at 25°C for the required time periods. The flasks were withdrawn from the bath at prefixed time intervals and the residual dye concentrations in the solutions were analyzed by centrifuging the mixtures and then measuring the absorbance of supernatants using a UV-visible spectrophotometer at the maximum wavelength of dye. The dye concentration was calculated by comparing absorbance to the dye calibration curve previously obtained.

### 2.4. Biosorption Data Evaluation

The dye biosorption amount of biosorbent, *q* (mg g^−1^), was calculated as [13]

(1) $$\begin{array}{c} \;q=\frac{\left(C_{o}-C_{t}\right)V}{M}, \end{array}$$

where *C*_*o*_ (mg L^−1^) is the initial dye concentration, *C*_*t*_ (mg L^−1^) is the residual dye concentration at time *t* (min), *V* (L) is the volume of dye solution, and *M* (g) is the amount of biosorbent used. The *q* value is equal to *q*_*t*_ at time *t* and *q*_*e*_ at equilibrium, respectively. In the same way, the *C*_*t*_ value is equal to *C*_*e*_ at equilibrium.

Each experiment for this biosorption study was repeated twice at the same conditions and the arithmetical average values obtained from these experiments were used to give the research results. In order to optimize the selected experimental factors based on Taguchi experimental design, the software Minitab (ver. 16.2.1, Minitab Inc., USA) was used. The parameters of kinetic and isotherm models with statistical evaluation data were defined by nonlinear regressions using the software OriginPro (ver. 8.0, OriginLab Co., USA).

## 3. Results and Discussion

### 3.1. Characterization of Biosorbent

**Figure 1:**
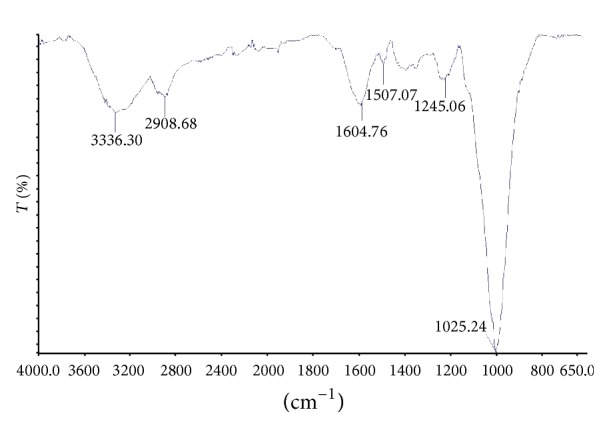
FTIR spectrum of biosorbent material.

Biosorption capacity of a biosorbent material depends upon porosity as well as chemical reactivity of functional groups at its surface [14]. Thus, knowledge of surface functional groups can give insight to the biosorption yield of the pine cone shell. The analysis of FTIR has played an important part in the investigation of biosorbent surface chemistry. Direct information on the presence of surface functional groups can be obtained from the infrared studies [15]. Pine cone is composed of epidermal and sclerenchyma cells which contain cellulose, hemicellulose, lignin, rosin, and tannins in their cell walls which contains polar functional groups such as alcohols, aldehydes, ketones, carboxylic, phenolic, and other groups [16]. These groups will form active sites for biosorption of dye on the material surface. The FTIR spectrum pattern for the pine cone shell is shown in Figure 1. Several peaks were observed from the spectrum indicating that the shell is composed of various functional groups which might be responsible for the dye biosorption. The spectra bands observed at 3336.30 and 2908.68 cm^−1^ represent –OH and aliphatic C–H groups, respectively [17]. The peak at 1604.76 cm^−1^ corresponds to the C=O stretch [18]. The peak at 1507.07 cm^−1^ may be due to the presence of aromatic rings [15]. The peak at 1245.06 cm^−1^is indicative of aliphatic acid group vibration due to deformation vibration of C=O and stretching formation of –OH of carboxylic acid and phenol [19]. The peak at 1025.24 cm^−1^ is associated with C–O–C functionalities [20].

**Figure 2:**
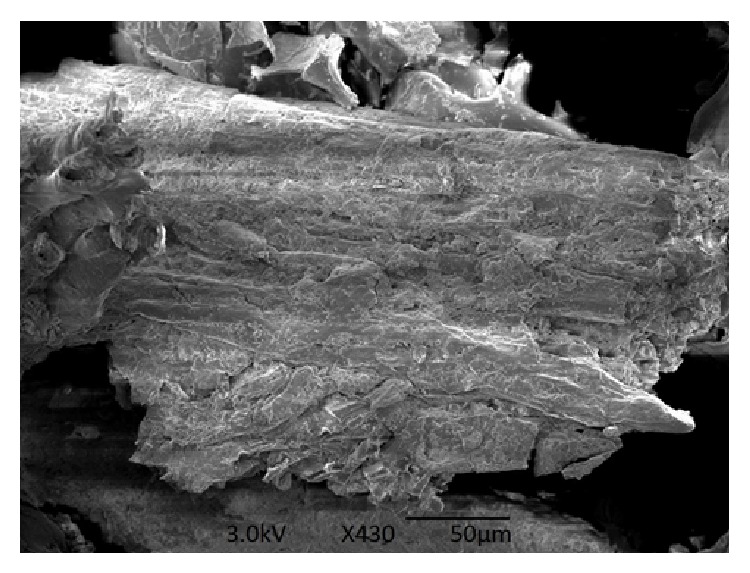
SEM picture of pine cone shell.

The analysis of SEM is a primary tool for characterizing the surface morphology of biosorbent material and fundamental physical properties of the biosorbent surface. It is useful for determining the particle shape, porosity, and appropriate size distribution of the biosorbent [21]. SEM picture of the pine cone shell is displayed in Figure 2. The figure clearly shows the presence of porous, rough, and irregular surface morphology of the biosorbent material. The cone shell has considerable numbers of cavities and pores. This is a good possibility for the dye molecules to be trapped and biosorbed [22].

### 3.2. Optimization Study of Biosorption System

The optimization of dye biosorption performance for the pine cone shell was performed using Taguchi experimental design. Taguchi L_9 _(3^3^) orthogonal array design containing the selected factors and their levels for this biosorption study is presented in Table 1. Taguchi design employs a generic signal-to-noise (SN) ratio as a quantitative measure for determining the optimum biosorption conditions. There are primarily three categories of SN ratios, namely, “smaller-is-better,” “larger-is-better,” and “nominal-is-best.” The selection principle of SN ratio depends on the goal of study. In order to maximize the biosorption of dye, the “larger-is-better” approach was adopted, in which the SN ratio was calculated by the following equation [23]:

(2) $$\begin{array}{c} \text{SN}\;\text{ratio}=-10\log\left(\frac{1}{n}\overset{n}{\underset{i=1}{\sum }}\frac{1}{y_{i}^{2}}\right), \end{array}$$

where *n* is the number of experiments and *y*_*i*_ is the value of dye biosorption capacity of each experiment. Based on the approach employed, the level of factor maximizing the SN ratio is optimal condition for the dye removal.

**Figure 3:**
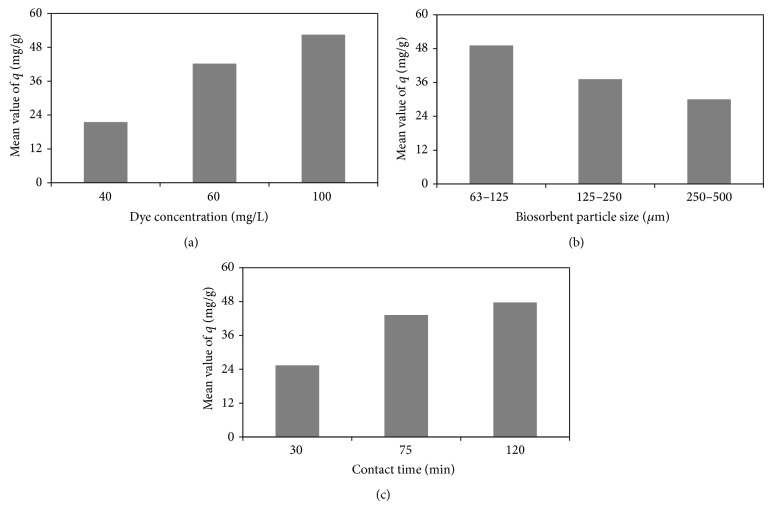
Effect of each factor studied on biosorption of dye.

The dye biosorption capacity as mean response and the value of SN ratio obtained for each experiment are given in Table 2. Besides, Table 3 and Figure 3 show the biosorption efficiencies and SN ratio values of all the levels of factors studied and the effect of each factor on the dye removal, respectively.  Figure 3(a) displays that the biosorption capacity of cone shell increased with increase in the initial dye concentration. This may be due to the high driving force for mass transfer at a high initial dye concentration. In addition, if the dye concentration in solution is higher, the active sites of biosorbent are surrounded by much more dye molecules and the biosorption occurs more efficiently [24]. As can be observed in Figure 3(b), the dye removal decreased with enhancing the biosorbent particle size. The higher dye biosorption efficiency with smaller particles can be due to the fact that smaller biosorbent particles provide a larger surface area and better accessibility of dye into active pores [25]. The biosorption capacity of pine cone shell increased with increase in contact time as shown in Figure 3(c). It may be attributed to more vacant active sites being available on the biosorbent surface for further dye biosorption until equilibrium [26]. According to the data presented in Table 3, the optimum dye biosorption conditions based on the approach adopted were obtained as the dye concentration of 100 mg L^−1^, biosorbent particle size of 63–125 *μ*m, and contact time of 120 min.

The analysis of variance (ANOVA) was performed to observe the effective factors and their confidence levels on the dye biosorption performance. From the results of ANOVA as given in Table 4, it was found that the dye concentration was the most effective factor studied on the removal of dye. Its contribution percentage was calculated to be 51.571%. This was followed by the contact time (28.739%) and biosorbent particle size (19.303%).

The test of confirmation is a crucial final step of Taguchi experimental design. Its purpose is to verify that the optimum biosorption conditions are suggested by the experimental design [27]. A verification experiment was performed based on the optimal dye removal conditions calculated previously. The predicted biosorption capacity for the pine cone shell from Taguchi design was obtained as 71.770 mg g^−1^ at the best biosorption conditions. The value was found to be 68.075 mg g^−1^ from the confirmation experiment. It is very close to the predicted performance. The results state that the experimental design is very effective.

**Figure 4:**
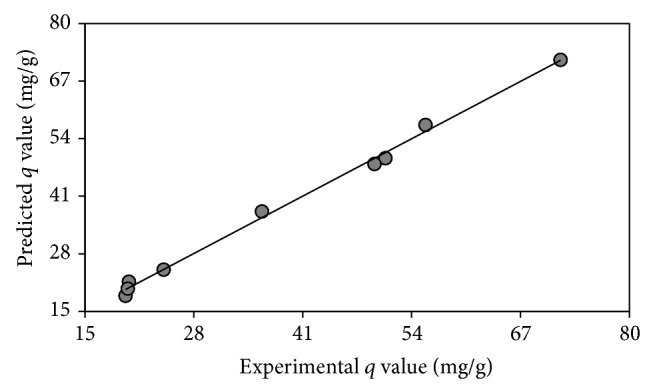
Comparison of experimental and Taguchi-predicted biosorption performance values.

A regression analysis was also carried out for comparison between the experimental and Taguchi-predicted biosorption performance values of the biosorbent for the biosorption test sets as observed in Figure 4. The determination of coefficient, *R* ^2^, was found to be 0.9961. This shows an excellent agreement between the experimental and predicted values. Thus, Taguchi experimental design provided reasonable predictive performance of dye biosorption.

### 3.3. Modeling of Biosorption Equilibrium

Biosorption isotherms describe how dye molecules interact with biosorbent material. They are critical for optimization of biosorption mechanism pathway, expression of surface property, and capacity of biosorbent and effective design of biosorption system [28, 29]. Thus, the equilibrium data obtained from the biosorption experiments were evaluated at the optimized dye removal conditions with Freundlich, Langmuir, and Dubinin-Radushkevich isotherm models.

Freundlich model assumes biosorptiononto heterogeneous solid surface and biosorption energy sites ofexponential type [30]. Based on the statistical information in Table 5, Freundlich model did not properly characterize the biosorption equilibrium. On the other hand, the value of *n*_*f*_ was found to be 3.3796 for C.I. Basic Red 46 biosorption by the pine cone shell. This represents a suitable biosorption [31].

**Figure 5:**
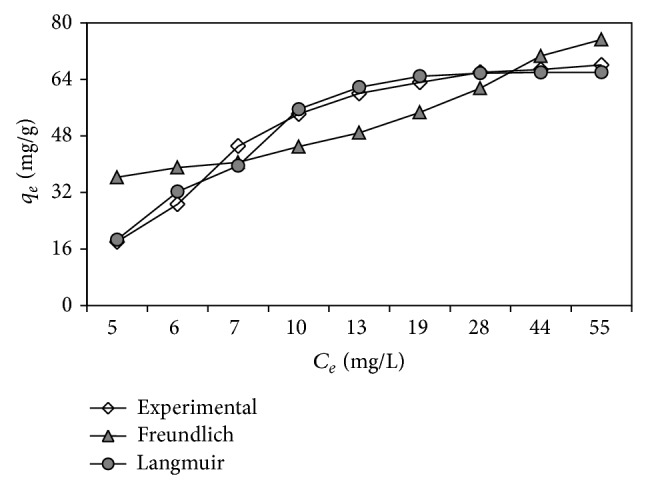
Plots for biosorption isotherm models.

Langmuir model proposes monolayer coverage and identical sites with the same biosorption energy on the biosorbent surface [32]. As can be seen in Table 5, with more suitable statistical results, Langmuir model fitted better to the biosorption data than Freundlich model. Furthermore, Figure 5 reveals that Langmuir model line was quite close to the experimental line during the biosorption period. This shows the monolayer coverage of C.I. Basic Red 46 dye molecules on the cone shell surface. On the other hand, for Langmuir-type biosorption system, the effect of isotherm shape on whether a biosorption process is favorable or unfavorable can be predicted by the separation factor, *R*_*L*_ [33]. The *R*_*L*_ value was obtained as 0.3861 for the removal of C.I. Basic Red 46 by the biosorbent. The values of *R*_*L*_ between 0 and 1 reflect a favorable biosorption [34].

Dubinin-Radushkevich model is generally applied to express the nature of biosorption as physical and chemical [35]. In Dubinin-Radushkevich isotherm, the mean free energy, *E* (kJ mol^−1^), shows the mechanism by which biosorption takes place [36]. A value of mean free energy below 8 kJ mol^−1^displays physical biosorption while a value between 8 and 16 kJ mol^−1^indicates chemical biosorption [37]. The mean free energy value for C.I. Basic Red 46 biosorption by the pine cone shell was found to be 3.2686 kJ mol^−1^ as shown in Table 5. This presents that the predominant mechanism of the biosorption of dye by the cone shell was likely physical biosorption. To support this information, the standard Gibbs free energy change, Δ*G*° (kJ mol^−1^), was determined by [38]

(3) $$\begin{array}{c} \Delta G^{{}^{\circ}}=-RT\ln\;K_{c}, \end{array}$$

where *K*_*c*_ is the distribution coefficient (*C*_*s*_/*C*_*e*_). *C*_*s*_ and *C*_*e*_ (mg L^−1^) are the equilibrium dye concentrations on biosorbent and in solution, respectively. The standard Gibbs free energy change for the biosorption of C.I. Basic Red 46 by the cone shell was calculated as −6.6536 kJ mol^−1^. A value of the change of free energy between −20 and 0 kJ mol^−1^ indicates a physical biosorption [39]. This result agrees well with that from the Dubinin-Radushkevich isotherm model.

### 3.4. Modeling of Biosorption Kinetics

Kinetic studies are important to understand the biosorption dynamics in terms of order of the rate constant. The kinetic parameters provide information for designing and modeling the biosorption process [40]. The data of biosorption kinetics for dye onto the biosorbent were analyzed under optimal biosorption conditions obtained with various kinetic models including the pseudo-first-order [40, 41], pseudo-second-order [42], logistic [43], and intraparticle diffusion [44]. As can be shown in Table 6, the pseudo-first-order was not an appropriate model for describing the biosorption kinetics based on the statistical evaluations. On the other hand, according to the statistical results presented in the table, the pseudo-second-order kinetic model provided a better fit to the experimental data obtained than the pseudo-first-order model. This confirms that the biosorption kinetics of dye onto the pine cone shellcan be accurately described by the pseudo-second-order model.

**Figure 6:**
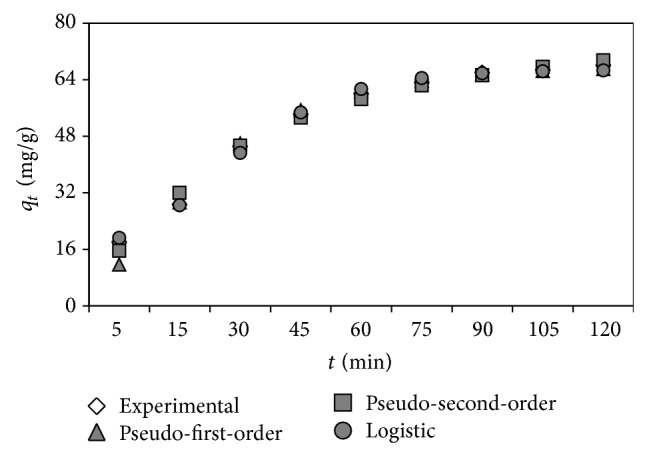
Comparison of kinetic models for biosorption dynamics.

The logistic model is mainly used for modeling of microbial growth and product formation [45, 46]. However, this model is slightly employed for explaining dye biosorption dynamics. The logistic model was used to define the biosorption kinetics of dye onto the cone shell and this model presented the best fit to the experimental results with the most suitable statistical outcomes as displayed in Table 6. Furthermore, Figure 6 shows that the logistic points were quite close to the experimental points over all the biosorption period. Thus, these results reveal that the logistic model can be applied effectively for characterizing the removal kinetics of C.I. Basic Red 46 by the pine cone shell.

The effect of intraparticle diffusion as a potential rate-controlling step in the biosorption was evaluated by Weber and Morris intraparticle diffusion model. According to this model, if a linear line passing through the origin exists between *q*_*t*_ and *t* ^1/2^, the intraparticle diffusion is the sole rate-limiting step. But, if multilinear plots are exhibited, two or more steps control the biosorption process [31]. The plot for dye biosorption by the biosorbent has three distinct regions (figure is not presented here). The initial region of the curve relates the biosorption on the external surface. The second stage corresponds to the gradual uptake presenting the intraparticle diffusion as rate-controlling step. The final plateau region indicates the surface biosorption and the equilibrium stage [24]. Hereby, the intraparticle diffusion was not the only rate-limiting step for the dye biosorption by the cone shell and also the other mechanism(s) may control the rate of biosorption or all of which may be operating simultaneously.

### 3.5. Assessment of Pseudo-Second-Order Kinetics Reflecting Biosorption Performance

An approaching equilibrium factor (*R*_*w*_) which represents the characteristics of kinetic curve of a biosorption system using the pseudo-second-order kinetic model was proposed by Wu et al. [47]. A family of curves for *R*_*w*_ = 0.01–1.00 can then be produced. When *R*_*w*_ = 1, the kinetic curve is called linear (zone 0). The possible causes of this effect are as follows: (i) it does not facilitate biosorption when the pseudo-second-order rate constant (*k*_2_) is very small, (ii) the equilibrium amount of biosorption (*q*_*e*_) is very small, and (iii) the longest operation time (*t*_*w*_) of biosorption process is too short. All these factors show an ineffective biosorption system where equilibrium cannot be reached. The curvature of biosorption curve increases as *R*_*w*_ reduces. The characteristic biosorption curve is called approaching equilibrium in the range 1 > *R*_*w*_ > 0.1 (zone I), called well approaching equilibrium in the range 0.1 > *R*_*w*_ > 0.01 (zone II), and called drastically approaching equilibrium when *R*_*w*_ < 0.01 (zone III). The value of approaching equilibrium factor for the biosorption of C.I. Basic Red 46 by the pine cone shell was found to be 0.0977 (Table 7). It lies in zone II under largely curved and well approaching equilibrium level. The characteristic curve obtained from graphical representation of approaching equilibrium factor can provide useful information under various operating conditions for effective biosorption system design [47].

Another parameter in the pseudo-second-order kinetic model which can reflect kinetic performance is the second-order rate index, *R*_*i*_ (min^−1^) [47]. The value of second-order rate index is equal to the inverse of biosorption half-life as shown in Table 7. The half-life of biosorption process, *t*_1/2_ (min), is the time for half amount of dye to be removed by the biosorbent [22, 48]. The value of half-life is suitable for facilitating the understanding of the operating time of a biosorption system. For the removal of C.I. Basic Red 46 by the cone shell, the half-life was obtained as 12.9945 min. The small half-life indicates the fast biosorption of dye onto the biosorbent [49]. On the other hand, the second-order rate index was found to be 0.0770 min^−1^ (Table 7). The second-order rate index is more suitable to describe the biosorption kinetics than the approaching equilibrium factor. The relationship between accurate operating time and amount of biosorption is an important factor in engineering practice. The second-order rate index is a key parameter affecting the fractional biosorption amount at any time [47]. The required operating times (*t*_*x*_) for various fractional biosorption amounts (*X*) for the biosorption of C.I. Basic Red 46 onto the cone shell are presented in Table 7. This information can be used to make proper decisions on scale up and design purposes [22]. Thus, from economic aspect, the most suitable fractional biosorption and operating time values should be specified based on actual operating conditions.

### 3.6. Dye Biosorption System Design

**Figure 7:**
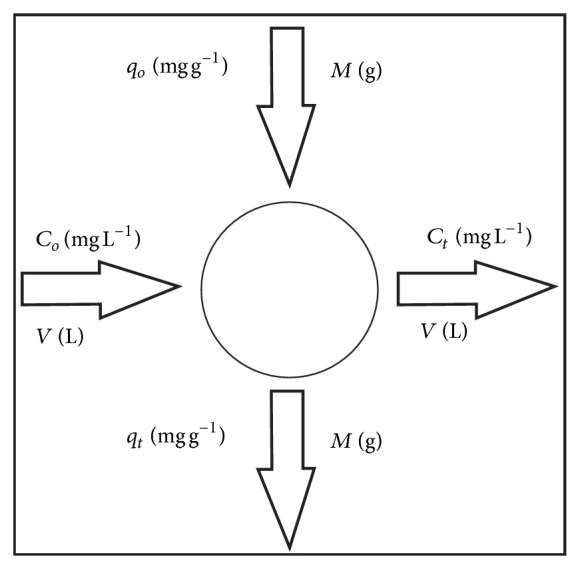
Design of single-stage batch system for dye biosorption.

Biosorption system or biosorber design based upon equilibrium isotherm data is a known technique for predicting the biosorber size and performance [17].  Figure 7 presents a schematic diagram for a single-stage batch dye biosorption system design where the starting inflow contains *V* (L) volume of dye solution and an initial dye concentration, *C*_*o*_ (mg L^−1^), which is to be reduced to *C*_*t*_ (mg L^−1^) in the biosorption process. In the treatment phase, a mass of *M* (g) biosorbent is added to this system and the dye loading on biosorbent changes from *q*_*o*_ to *q*_*t*_ (mg g^−1^). The mass balance for dye in single-stage batch dye biosorption is given by [50, 51]

(4) $$\begin{array}{c} V\left(C_{o}-C_{t}\right)=M\left(q_{t}-q_{o}\right)=Mq_{t}. \end{array}$$

Langmuir isotherm presents better fit to the equilibrium data for C.I. Basic Red 46 biosorption by the cone shell. Thus, the mass balance based on Langmuir model under equilibrium (*C*_*t*_ → *C*_*e*_ and *q*_*t*_ → *q*_*e*_) can be obtained by rearranging (4) as

(5) $$\begin{array}{c} \frac{M}{V}=\frac{C_{o}-C_{e}}{q_{e}}=\frac{C_{o}-C_{e}}{q_{L}bC_{e}/\left(1+bC_{e}\right)}. \end{array}$$

**Figure 8:**
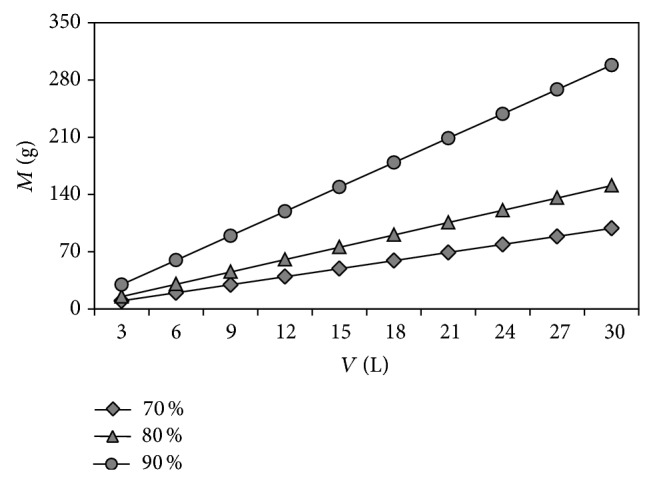
Required biosorbent amount (*M*) versus volume of dye solution treated (*V*).

The biosorbent amount required to achieve a specific dye removal percentage at a given dye solution volume can be predicted using (5). For different removal percentages of C.I. Basic Red 46 dye, a series of plots of *M* versus *V* is shown in Figure 8 at optimum biosorption conditions previously obtained by Taguchi experimental design. For example, the required amount of the pine cone shell for 80% dye removal is 105.4672 g for dye solution volume of 21 L. Thus, a design procedure for a single-stage batch dye biosorption system is outlined and this information can be useful for the application of the cone shell on a large scale for the dye removal.

## 4. Conclusion

The dye biosorption performance for the pine cone shell was successfully optimized using Taguchi experimental design model. This model provided reasonable predictive performance of dye biosorption (*R* ^2^: 0.9961). The dye concentration had the most significant impact on the dye removal with 51.571% contribution. Langmuir model fitted better to the biosorption data than Freundlich model. This showed the monolayer coverage of dye molecules on the biosorbent surface. The nature of biosorption of dye by the biosorbent was likely physical biosorption based on Dubinin-Radushkevich isotherm model and the standard Gibbs free energy change. The logistic model was found suitable in describing the biosorption kinetics. The kinetic parameters reflecting biosorption performance from the pseudo-second-order kinetics revealed an effective dye biosorption system. A design procedure for a single-stage batch dye biosorption system was also outlined. The study showed that the pine cone shell can be an efficacious biosorbent in the dye removal from water.

## Figures and Tables

**Table 1:** Taguchi L_9_ (3^3^) orthogonal array design.

Experiment	Operating factors		
	Dye concentration (mg L^−1^)	Biosorbent particle size (*µ*m)	Contact time (min)
1	40	63–125	30
2	40	125–250	75
3	40	250–500	120
4	60	63–125	75
5	60	125–250	120
6	60	250–500	30
7	100	63–125	120
8	100	125–250	30
9	100	250–500	75

**Table 2:** Dye biosorption capacity and SN ratio value obtained for each experiment.

Experiment	Mean response	
	Dye biosorption capacity. *q* (mg g^−1^)	SN ratio
1	18.5075	25.2275
2	24.3769	28.1887
3	21.6554	26.1722
4	57.0611	34.9478
5	49.5265	33.6339
6	20.1080	26.5140
7	71.7701	37.5616
8	37.5385	31.1443
9	48.2210	33.4029

**Table 3:** Biosorption efficiencies and SN ratio values obtained for all levels of factors.

Factor	Level	Biosorption efficiency. *q* (mg g^−1^)	SN ratio
Dye concentration (mg L^−1^)	40	21.51	26.53
	60	42.23	31.70
	100	52.51	34.04

Biosorbent particle size (*µ*m)	63–125	49.11	32.58
	125–250	37.15	30.99
	250–500	29.99	28.70

Contact time (min)	30	25.38	27.63
	75	43.22	32.18
	120	47.65	32.46

**Table 4:** Results of analysis of variance (ANOVA).

Factor	Degree of freedom	Sum of squares	Mean squares	Fischer ratio	*P* value	Percent contribution
Dye concentration	2	1495.69	747.843	133.09	0.007	51.571
Biosorbent particle size	2	559.83	279.917	49.82	0.020	19.303
Contact time	2	833.50	416.748	74.17	0.013	28.739
Error	2	11.24	5.619			0.388

Total	8	2900.25				

**Table 5:** Data of isotherm models for dye biosorption.

Model	Equation	Parameter	Value	*R* ^2^	SD
Freundlich	*q*_e_ = *K*_f_ *C*_e_^1/*n*_f_^	*K*_*f*_ *n*_*f*_	23.0384 3.3796	0.6823	10.8889

Langmuir	$q_{e}=\frac{q_{L}bC_{e}}{1+bC_{e}}$ $R_{L}=\frac{1}{1+bC_{o}}$	*q*_*L*_ *R*_*L*_	66.0207 0.3861	0.9782	3.0842

Dubinin-Radushkevich	*q*_e_ = *q*_DR_exp⁡^−*B*_DR_*ε*^2^^ $E=\frac{1}{{\left(2B_{\text{DR}}\right)}^{1/2}}$	*q*_DR_ *E*	69.5042 3.2686	0.9654	3.5914

SD: standard deviation, *K*_*f*_ (mg g^−1^) (L mg^−1^)^1/n^: a constant related to biosorption capacity, *n*_*f*_: a constant related to biosorption intensity, *q*_*L*_ (mg g^−1^): maximum monolayer biosorption capacity, *b* (L mg^−1^): a constant related to energy of biosorption, *R*_*L*_: separation factor, *q*_DR_ (mg g^−1^): maximum biosorption capacity, *B*_DR_ (mol^2^ kJ^−2^): a constant related to mean free energy of biosorption, *ε*: Polanyi potential, and *E* (kJ mol^−1^): mean free energy.

**Table 6:** Kinetic parameters of dye removal.

Model	Equation	Parameter	Value	*R* ^2^	SD
Pseudo-first-order	*q*_t_ = *q*_e_(1 − exp⁡^−*k*_1_*t*^) *h*_1_ = *k*_1_ *q*_e_	*k*_1_	0.0378		
		*q*_*e*_	67.7251	0.9827	2.5403
		*h*_1_	2.5580		

Pseudo-second-order	$q_{t}=\frac{k_{2\;}{q_{e}}^{2}t}{1+k_{2\;}q_{e}t}$ *h*_2_ = *k*_2_ *q*_e_^2^	*k*_2_	0.0011		
		*q*_*e*_	69.9680	0.9908	1.9973
		*h*_2_	5.3844		

Logistic	$q_{t}=\frac{q_{e}}{1+\exp^{-k(t-t_{c})}}\;$	*q*_*e*_	66.7553		
		*k*	0.0605	0.9957	1.3725
		*t*_*c*_	19.9207		

Intraparticle diffusion	*q*_t_ = *k*_p_ *t* ^1/2^ + *C*	*k*_*p*_	5.93564	0.9431	4.6093
		*C*	9.08756		

SD: standard deviation, *k*_1_ (min^−1^), *k*_2_ (g mg^−1^ min^−1^), and *k*_*p*_ (mg g^−1^ min^−1/2^): biosorption rate constants, *h*_1_ and *h*_2_ (mg g^−1^ min^−1^): initial biosorption rates, *k* (min^−1^): maximum relative biosorption rate, *t*_*c*_ (min): time *t* pointing center of *q*_*e*_ (*q*_*e*_/2), and *C* (mg g^−1^): a constant providing information about thickness of boundary layer.

**Table 7:** Dye biosorption performance data based on pseudo-second-order kinetics.

Parameter	Symbol (unit)	Equation	Value
Approaching equilibrium factor	*R*_*w*_ (—)	$R_{w}=\frac{1}{1+k_{2}q_{e}t_{w}}$	0.0977
Second-order rate index	*R*_*i*_ (min^−1^)	*R*_i_ = *k*_2_ *q*_e_	0.0770
Biosorption half-life	*t*_1/2_ (min)	$t_{1/2}=\frac{1}{k_{2}q_{e}}$	12.9945
Operating time	*t*_*x*_ (min)	$t_{x}=\frac{W}{k_{2}q_{e}}$	
*t*_0.75_			38.9835
*t*_0.85_			73.6356
*t*_0.95_			246.8958

*t*_*w*_ (min): longest operation time based on kinetic experiments, *X*: fractional biosorption value, and *W*: *X*/(1 − *X*).
